# Lipid metabolism and storage in neuroglia: role in brain development and neurodegenerative diseases

**DOI:** 10.1186/s13578-022-00828-0

**Published:** 2022-07-12

**Authors:** Danying Yang, Xifeng Wang, Lieliang Zhang, Yang Fang, Qingcui Zheng, Xing Liu, Wen Yu, Shoulin Chen, Jun Ying, Fuzhou Hua

**Affiliations:** 1 Department of Anesthesiology, the Second Affiliated Hospital of Nanchang University, Nanchang, 330006 Jiangxi China; 2 Key Laboratory of Anesthesiology of Jiangxi Province, 1# Minde Road, Nanchang, 330006 Jiangxi People’s Republic of China; 3 Department of Anesthesiology, the First Affiliated Hospital of Nanchang University, Nanchang, 330006 Jiangxi China

**Keywords:** Neuroglia, Cholesterol, Sphingolipids, Fatty acids, Lipid droplets, Neurodegeneration

## Abstract

The importance of neuroglia in maintaining normal brain function under physiological and pathological conditions has been supported by growing evidence in recent years. The most important issues regarding glial metabolism and function include the cooperation between glial populations and neurons, morphological and functional changes in pathological states, and the role in the onset and progression of neurodegenerative diseases. Although lipid accumulation and further lipid droplet production in neurodegenerative disease brain models have been observed for a long time, the dynamic development of brain lipid droplet research in recent years suggests its role in the development and progression of neurodegenerative diseases was previously underestimated. First recognized as organelles of lipid storage, lipid droplets (LDs) have emerged as an important organelle in metabolic diseases, inflammation, and host defense. Dynamic changes in lipid metabolism within neurons and glial cells resulting in lipid accumulation and lipid droplet formation are present in brain models of various neurodegenerative diseases, yet their role in the brain remains largely unexplored. This paper first reviews the metabolism and accumulation of several major lipids in the brain and discusses the regulation of lipid accumulation in different types of brain cells. We explore the potential role of intracellular lipid accumulation in the pathogenesis of neurodegeneration, starting from lipid metabolism and LDs biogenesis in glial cells, and discuss several pathological factors that promote lipid droplet formation, mainly focusing on oxidative stress, energy metabolism and glial cell-neuron coupling, which are closely related to the etiology and progression of neurodegenerative diseases. Finally, the directions and challenges of intracellular lipid metabolism in glial cells in neurodegeneration are discussed.

## Introduction

The dynamic interactions between glial cells and neurons maintain the normal functioning of the brain. Neurodegenerative diseases are characterized by progressive neuronal dysfunction and death, and neuronal survival depends on the support of neuroglia, which is vulnerable to pathological factors such as neuroinflammation, oxidative stress, and aging. As the second most lipid-rich organ, lipid metabolism in the brain is closely linked to brain energy homeostasis, oxidative stress, and neuroinflammation, and imbalances in neuroglial cell lipid metabolism affect normal neuronal activity.

The brain contains large amounts of sphingolipids and cholesterol, which are involved in synaptogenesis and neurogenesis, impulse transmission, and are inextricably linked to the development and maintenance of the brain and the proper conduct of many other cellular processes [1, 2]. However, lipid accumulation production of lipotoxic metabolites induced by impaired lipid metabolism in the brain may further trigger central nervous system diseases and injuries. LDs are independent organelles associated with lipid storage, for example, excess free cholesterol (FC) is converted to cholesteryl esters (CE) and stored in LDs, and ceramides are separated into acyl ceramides for storage in LDs. For a long time, LD formation was considered to be primarily associated with lipid transport, serving as relatively simple storage particles for lipid. Newly emerged research is starting to shed light on the functions of LDs in pathophysiology.

The histological presence of LDs in human brain glial cells has been discovered over 100 years ago [1]. Synthesis and storage of neutral lipids is a fundamental property of eukaryotic cells, and most eukaryotic cell types store fat in cytoplasmic LDs, culminating in the compartmentalization of lipids. While the brain is under pathological conditions, the content of LDs in glial cells is increased, but to a lesser extent in neurons. It has been controversial whether this lipid accumulation under stress has a protective or detrimental role on the brain. Disruption of lipid metabolism and energy homeostasis are prevalent in neurodegenerative diseases such as amyotrophic lateral sclerosis (ALS) [3]. In addition, LDs are thought to be the initial sites of α-synuclein aggregation in patients with Alzheimer's disease (AD) and Parkinson's disease (PD) [2].

LDs are highly regulated organelles that contribute to many cellular processes and emerge as metabolic hubs with diverse roles in energy and signaling precursors storage, cell stress management, and protein handling [4, 5]. LDs accumulation became of interest in all brain cell types when LDs in glia were discovered to contribute to neurodegeneration. Almost all brain cells have been found to contain LDs, and LD accumulation in microglia and astrocytes is the most studied. Interestingly, LD-rich microglia exhibit a different pathology-related functional phenotype, with genes regulating LD production overlapping with some genes that cause autosomal dominant neurodegeneration (SLC33A1, SNX17, VPS35, CLN3, NPC2, and GRN) [6]. Although a link between LDs and neurodegeneration has been demonstrated, the role of LDs in the brain is relatively unstudied.

In this review, we describe several important lipids in the brain, summarize the current pathological correlates affecting neuroglial LD accumulation, the relevance of several degenerative diseases to LDs, and focus on the potential impact of neuroglial cell lipid dynamics on the development of degenerative brain lesions.

## Lipid synthesis and function in the brain

### Cholesterol

Cholesterol is a major component of cell membranes and is important for the development and function of the brain, regulating membrane fluidity and synaptogenesis. Cholesterol in the brain is mainly distributed in myelin sheaths formed by oligodendrocytes and cell membranes of glial cells and neurons. The formation of synapses requires large amounts of cholesterol, and there is substantial evidence that cholesterol is widely distributed in pre- and postsynaptic regions to maintain and organize synaptic proteins, decreased synaptic transmission and impaired synaptic plasticity were observed in cholesterol-deficient neuronal cells [4, 7]. The cholesterol requirement for neurite regrowth increases in a variety of neuronal types after nerve injury, the cholesterol-rich transporter lipoprotein Apo-E accumulates at regenerating axon sites [5], and efficient cholesterol transport also plays a critical role in nerve regeneration.

Cholesterol in the brain is mainly derived from de novo synthesis due to the presence of the blood–brain barrier (BBB). The rate of cholesterol synthesis peaks early in human and rodent brain development and during myelin formation, after which brain cholesterol synthesis and consumption remain at very low levels and its steady-state concentration remains essentially constant [8]. Although differences in cholesterol biosynthesis among brain cells are controversial, several studies suggest that cholesterol production, transport, and internal environmental stability in the brain are very different during different periods.

Developing neurons have an active de novo cholesterol synthesis and produce significantly higher amounts of cholesterol than astrocytes [3]. By comparing the differences in cholesterol precursors in neurons and astrocytes, it was found that neurons contain mainly precursors of the Kandutsch-Russel pathway, including lanolin (LA), whereas astrocytes contain desmosterol, a precursor of the Bloch pathway, indicating that neurons and astrocytes use different routes for cholesterol synthesis. Compared to astrocytes, adult neurons have very low levels of the squalene precursor converting enzymes lanosterol-converting enzymes-24-dehydrocholesterol reductase (DHCR24) and lanosterol 14-alpha demethylase (CYP51) [6], which struggle to efficiently convert LA and exhibit lower rates of cholesterol synthesis. A series of studies on rat retinal ganglion cells (RGCs) have shown that in the absence of glial cells, neurons form immature synapses with low numbers and inefficient transmission. This study also indicates that synaptogenesis is limited by glial-derived cholesterol availability, consistent with the fact that most synapses in the developing brain are formed after macroglia differentiation [9].

Dysregulation of cholesterol metabolism is associated with a variety of neurodegenerative pathologies, and AD is the most studied. Excess FC is converted to CE by cholesterol acyltransferase (ACAT) and subsequently accumulated in intracellular LDs or effluxed to the extracellular environment [10]. Increased cholesteryl-ester levels enhance the release of β-amyloid(Aβ) in cultured cells, while ACAT1 gene ablation allows the conversion of excess free brain cholesterol into 24(S)-hydroxycholesterol to cross the BBB and reach the periphery [11, 12]. In vivo, ablation of the ACAT1 gene in 3XTg-AD mice leads to reduced hAPP and HMGR protein levels and ameliorates amyloid pathology [12]. Although the molecular mechanism between cholesterol metabolism and the pathological process of AD is unclear, current evidence indicates that the balance between FC and CE is a key parameter in the control of amyloid production.

### Sphingolipids

As with cholesterol, sphingolipids (SP) are the major lipids that compose the brain and the functional units of neuronal cell membranes and are highly enriched in the nervous system. Although the types of SP that may contribute to neurological dysfunction are unknown, genetic defects in various sphingolipid metabolizing enzymes reveal the importance of sphingolipid metabolism in brain development and health [13]. SP are a class of lipids featured with a sphingosine backbone, this class of lipids exhibits great diversity and complexity, and SP shows different biological activities due to the variability of their functional groups. Major bioactive SP include ceramide, sphingomyelin, and Sph-1-phosphate (S1P), some of which function as bioactive molecules involved in the regulation of cell growth, differentiation, senescence, and apoptosis [14]. Sphingolipid metabolism is associated with a variety of neurological disorders, it is involved in myelin stability, and neuron-glial connections, and is related to neuronal differentiation and synaptic transmission [15].

Ceramide is a central point of biosynthesis and catabolism, which can be produced via the salvage pathway and the de novo pathway, both controlled by the ceramide synthase (CerS). Attachment of various head groups at the C1 position of ceramide can form the basic structural unit of more complex SP, and degradation of complex SP contributes to the formation of a ceramide pool that can be reused for complex sphingolipid synthesis or catabolism [16]. Ceramide is raised in the human brain during AD and is considered to be one of the predictive serum biomarkers [17, 18]. Recent studies have demonstrated that the fatty acyl CoA synthase ACSL5 cooperates with all CerS to isolate biologically active ceramide into acyl ceramide and store them in LDs. This pathway, catalyzed by diacylglycerol acyltransferase 2 (DGAT2) on LDs, leads to elevated acyl ceramide synthesis and storage which may be a protective mechanism that attenuates ceramide-mediated apoptosis [19].

Sphingomyelin is hydrolyzed by neutral sphingomyelinase-2 (nSMase) in the hippocampus to ceramide. Inhibition of SMS2 promotes neuronal exosome secretion and may be involved in neurotoxic amyloid-β clearance by microglia [20]. Sphingomyelin can also be reconverted to ceramide by CerS or by dephosphorylation by sphingosine kinase, resulting in S1P. S1P is the most studied bioactive sphingolipid, which can be secreted by cells as a signaling molecule and bind to G protein-coupled receptors and can also act as a regulatory factor in regulating histone acetylation within cells [21]. In addition, S1P is a key mediator of immune function [22], but its function in the brain has not been elucidated. Recent studies have demonstrated that S1P accumulation in neuronal cells induces alterations in microglia. Kilunakaran et al. observed S1P accumulation in astrocytes after knockdown of S1P lytic enzyme SGPL1 in neuronal cells, followed by microglia activation and increased expression of pro-inflammatory mediators TNF and IL-6 [23].

### Free fatty acids (FFAs)

Fatty acids (FAs) are a component of cell membrane phospholipids and a fuel for oxidative phosphorylation. As we know, FAs can enter the brain and undergo oxidative degradation, and in nutrient-deficient states, cellular energy supply shifts from dependence on glucose metabolism to dependence on mitochondrial FA oxidation [24], a recent study has shown that fatty acid oxidation accounts for approximately 20% of the total energy consumption of the human brain [25]. FFAs trigger a variety of harmful activities in the cell, active neuronal cells produce excessive FAs without being able to utilize hydrogen-rich fatty acids to promote oxidative adenosine triphosphate (ATP) synthesis, resulting in the accumulation of toxic FAs in neurons, which are required to be stored in intracellular LDs as triglycerides to avoid neuronal damage. Excess FAs are transported by apolipoproteins into astrocytes, which are rich in LDs and less susceptible to harmful ROS activity than neurons and are thought to be the main sites of FAs storage and metabolism in the brain [25]. In terms of energy supply, LDs act as energy storage stations, transporting FAs to mitochondria during nutrient depletion and being consumed as an alternative energy source. Thus, to protect neurons from FFA-related lipotoxicity and to meet the energy supply in specific situations, FA storage and oxidation processes seem to depend on a close metabolic link between neurons and astrocytes [26] Lipid peroxidation levels are elevated in brain and body fluids in a variety of neurodegenerative disease models, particularly AD, PD, ALS, and Huntington’s disease (HD) [27–30]. Stimulation of neuronal excitotoxicity increases lipid and FA peroxidation, which are toxic and disrupt the integrity of mitochondria, leading to mitochondrial dysfunction. If hyperactive neurons are incapable of consuming or removing these peroxidized FAs, they will undergo pathophysiology that will eventually lead to neurodegeneration [31].

## Lipid metabolism and storage in the neuroglia

### Lipid metabolism in astrocytes

Astrocytes are morphologically complex and prevalent neuroparenchymal cells in the nervous system. Due to the multiple roles of astrocytes in supporting neuronal structure and survival, the imbalance of brain lipid homeostasis and impaired energy transduction caused by astrocytes has been the focus of research on the pathology of various brain diseases. Cholesterol may play the most critical role in the structure of astrocytes among all the lipids present in astrocytes. Among the subtypes of glia, Oligodendrocytes produce cholesterol for myelin formation, which is involved in brain maturation and neurotransmission, while astrocytes are thought to be the main site of exogenous neuronal cholesterol synthesis [32]. Neuroglia has been shown to secrete lipoproteins in vivo and to secrete lipoprotein particles as carriers in the form of cholesterol-Apo-E complexes outside the cell, providing large amounts of cholesterol for synaptogenesis [9]. Beyond this, there is growing evidence supporting a possible role of astrocytes in regulating myelin formation, and whether or to what extent astrocyte-derived cholesterol is involved in myelin formation remains to be investigated.

Astrocytes are the predominant cell type in the hippocampus that expresses the sterol regulatory element-binding protein (SREBP), and astrocyte-restricted inactivation of SCAP-SREBP-mediated lipid biogenesis in mice suggests that reduced SREBP activity in astrocytes leads to impaired presynaptic terminal development and hippocampal function, presumably through a reduction of presynaptic protein SNAP-25 levels and the number of synaptic vesicles [33]. Moreover, inactivation of the major transcriptional regulator of the cholesterol synthesis gene SREBP2 in astrocytes results in reduced brain size in mice, particularly in astrocyte-rich regions [34]. ACAT1/SOAT1 is activated in astrocytes under conditions such as excessive cholesterol content or lack of Apolipoprotein E (ApoE) and exogenous, leading to enhanced lipid storage processes [35].

Ceramide in astrocytes may contribute to the pathogenesis of several neurodegenerative diseases, mainly by promoting neuroinflammation and neuronal apoptosis [17, 36]. Using a combination of proteomic, metabolomic, transcriptomic, and perturbation studies, Chao and colleagues found that sphingolipid metabolism in astrocytes triggers the interaction between the C2 structural domain in cytoplasmic phospholipase A2 (cPLA2) and the CARD structural domain in mitochondrial antiviral signaling protein (MAVS), thereby facilitating an NF-κB-driven transcriptional program that promotes central nervous system (CNS) inflammation in experimental autoimmune encephalomyelitis (EAE) and multiple sclerosis [37]. In addition, S1P signaling is an important target associated with the control of infiltration of peripheral immune cells into the CNS, and S1P receptor expression is upregulated in astrocytes in demyelinating and chronic multiple sclerosis (MS) lesions. Selective knockdown of S1P signaling in astrocytes reduces the severity of EAE, demyelination, and axonal loss [38].

### Lipid metabolism in microglia

To ensure the accurate and stable function of neurons, brain metabolism is tightly controlled. As guardians of the central nervous system, microglia express multiple receptors to perceive variations in interstitial lipid composition [39]. The expression of an essential phagocytic receptor CD36 on the surface of microglia mediates the uptake of myelin debris by microglia, and myelin internalization increases CD36 expression via NRF2, enhancing secondary uptake of myelin, thus establishing an important and interesting positive feedback process [40]. The triggering receptor expressed on myeloid cell 2 (TREM2) is another immune receptor expressed in microglia that functions by sensing lipids and mediating myelin phagocytosis. TREM2-deficient microglia phagocytose myelin debris but are incapable of removing myelin cholesterol, resulting in the accumulation of pathogenic CE in microglia [41].

As the major phagocytic cells in the brain, microglia are thought to play a role in the clearance of Aβ. Aβ binds to lipoproteins (ApoE and CLU) and this complex is internalized by microglia in a TREM2-dependent manner, and recognition of these ligands by TREM2 variants is associated with AD risk is significantly reduced or even abolished [42]. After internalizing myelin by microglia via multiple surface receptors, for example, Low-density lipoprotein receptor-related protein 1(LRP1) [43], myelin degradation in lysosomes generates free FAs that can be used as components of membranes, stored in LDs, oxidized and catabolized, or exported via the efflux system. Notably, lipid accumulation in microglia also triggers lipid response signaling pathways. The liver X receptors (LXRs) are a cholesterol sensor that controls intracellular and systemic cholesterol homeostasis, and myelin contains ligands that activate LXRβ, thereby increasing the expression of ABCA1 and ApoE, and further enhancing microglia clearance activity [44, 45]. In the regulation of intracellular lipid processing, cellular compartments interact through membrane contact sites (MCS), which interconnect mitochondria with multiple nodes of the endosomal system, and disturbances in any of these processes promote alterations in microglia liposomes and functions that directly affect the maintenance of homeostasis in the brain [46].

Microglia containing intracellular myelin remnants are one of the pathological hallmarks of MS, and myelin overload within microglia may affect CNS repair and neuroinflammation by inducing abnormal immune responses and damaging tissue regeneration. Similar to astrocytes, dysfunction of transporter proteins also facilitates lipid deposition in microglia. Cholesterol transport defects in apolipoproteins accelerate the accumulation of CE in microglia during aging and demyelination [47]. Monounsaturated fatty acid production by Stearoyl-CoA desaturase-1 (SCD1) impairs ABCA1 transporter-mediated cholesterol efflux and sustained intracellular accumulation of myelin inhibits phagocytic repair, while conversely, depletion of SCD1 prevents myelin-induced phenotypic transformation and promotes myelin regeneration [48]. Although numerous in vivo and in vitro experiments have demonstrated that defects in myelin processing are associated with disease progression [49–51], the causal relationship between lipid accumulation in microglia inflammatory phenotypic transition and myelin destruction remains controversial and clarifying the actual triggers of lipid accumulation in microglia is essential to elucidate demyelinating disease pathology.

### Lipid droplet synthesis and decomposition

First recognized as organelles of lipid storage, LDs have emerged as an important organelle in metabolic diseases, inflammation, and host defense. The core of LDs contains hundreds of species of neutral lipids, in most cell types, primarily comprising triacylglycerols (TG), CE, and sterol esters (SEs). The procedure of neutral lipid composition and storage in LDs protects cells from lipotoxicity caused by excessive lipid accumulation [52, 53]. Neutral lipid synthesis is a sophisticated process in which multiple related proteins cooperate. Despite the exciting advances in LD generation in recent years, the exact mechanisms remain to be investigated.

LD assembly involves several discrete steps, beginning with the buildup of neutral lipids between ER bilayers. It uses glycerolphosphate and fatty acyl-CoA as its raw materials to synthesize glycerolipids, as in glycerophospholipids or TGs [54]. This pathway is the main way of TG composition in most cells and is known as the de novo glycerolipid synthesis pathway. Each step of neutral LD formation is catalyzed by a different enzyme, and DGAT catalyzes the last step of each TG formation pathway and is important in LD synthesis. DGAT1 and DGAT2 are two enzymes with non-overlapping functions, and they jointly regulate LD biogenesis, while DGAT1 is specifically required for LD production under starvation [55]. Both DGAT1 and DGAT2 have functions implicated in mitochondria, DGAT2 was found to co-localize with the attachment of LD surface and mitochondria, and it may play a role in facilitating the binding of LDs to mitochondria [56]. DGAT1 transports autophagy-liberated FAs to LDs that are close to mitochondria, avoiding impairment of mitochondrial function [57]. Whether LDs are in contact with mitochondria or not, the two are indeed inextricably linked in terms of metabolism and function, which we will discuss in detail in the later section. Lipid flows from the ER to LDs when sufficient neutral lipids accumulate within the ER bilayer. After budding from ER, LD growth and expansion, which through the transfer of TAG to LDs via membrane bridges between the lipid and ER or through the fusion of two LDs into one LD [58]. Although the initial budding of LDs is a biophysical process, which can be regulated by surfactants and may also be assisted by specific proteins such as FIT2 [59].

LDs are encircled by a polar lipid monolayer with several decorating proteins, some of which are linked to lipid metabolism, known generically as perilipins (PLINs) in mammalian LDs [60, 61]. The perilipin family is the best-studied of the LD-associated proteins, Sztalryd and colleagues described PLINs aptly as “gatekeepers” of intracellular lipolysis [62]. There are PLIN1-5 in humans, they are thought to contribute to the formation of LDs by protecting them from lipase catabolism. PLIN-2 regulates intracellular lipid metabolism through the PPARalpha/RXRA and CREB/CREBBP signaling pathways, and overexpression of PLIN-2 protects LDs from lipolysis in a variety of cells [63]. Similarly, as LDs scaffolding proteins, PLINs may also play a role in the interaction of LDs with mitochondria, for example, PLIN-5 expressing cells show decreased LD hydrolysis and control local FA flux to protect mitochondria.Degradation of LDs generally appears in the form of lipophagy or lipolysis, and this progress is highly regulated by the protein composition on the surface of LDs. Underfed conditions, lipolysis is activated by LD-associated lipases, such as adipose triglyceride lipase (ATGL), hormone-sensitive lipase (HSL), and monoglyceride lipase (MGL), which promote the breakdown of TAG to FAs. ATGL is the rate-limiting enzyme for LD-associated triglyceride hydrolysis, and activation of ATGL is the first step of lipolytic hydrolysis, which then catalyzes the TAG hydrolysis to diacylglycerol (DAG) and FA. DAG subsequently acts as the substrate for HSL, a multifunctional enzyme, which converts it to monoacylglycerol (MAG) and FA. At the last of the process, MAGs are released into the cytosol and cleaved by MGL to glycerol and FA.

Typically, autophagy is one of the major degradation pathways for many organelles that enables them to survive and renew under stress. TAG stored in LDs breakdown through an autophagic process termed lipophagy, a form of macroautophagy, which was discovered in 2009 [64]. During lengthy fasting, this lysosomal–autophagic process plays an important role in lipid degradation. Initiating by sequestering LDs in a double membrane vesicle, then LDs can be delivered to lysosomes for degradation via actions of lytic enzymes and mobilized to generate free FAs. In non-adipocytes such as neurons, mitochondria or peroxisomes convert FA from the bloodstream to acetyl-CoA via β-oxidation, which can boost the citric acid cycle and produces ATP.

## Pathological factors that promote neuroglial lipid storage

Under physiological conditions, cargo-specific clearance of organelles by selective autophagy is important for maintaining the stability of the neuronal internal environment. Lipids are the main components of cell membranes, during periods of prolonged starvation, lipids released by macroautophagy/autophagy breakdown of membrane organelles are packaged and stored in new LDs [55]. Furthermore, LDs formation occurs in a variety of pathological processes and LDs may have different functional phenotypes in different situations or different cell types.

### Neuroinflammation and oxidative stress

Inflammation is an important immune response of physiological origin, glial cells act as innate immune cells in the central nervous system, and the joint action of multiple glial cells and peripheral immune cells triggers neuroinflammation. Chronic inflammation of the CNS is one of the contributing factors to a range of neurodegenerative disease pathological processes. Microglia are the most active group of cells in the central nervous system, which are activated by inflammation, thereby performing phagocytosis to counter inflammation. Recent studies have identified a new type of LD accumulating microglia (LDAM) in the hippocampus of aging mice. This novel identified microglial cell types have a reduced phagocytic capacity and even releases inflammatory mediators that promote age-related inflammation [65]. In this experiment by Marschallinger, lipopolysaccharide (LPS) was used to induce LDAM production, suggesting that it may be aging-related neuroinflammation that leads to the accumulation of LDs in microglia. However, the causal relationship between inflammatory factors and lipid droplet production was not elucidated in this experiment.

Proteins with a well-defined role in the pathogenesis of inflammation have been demonstrated to compartmentalize within LDs in a variety of cell types [66]. LDs contain enzymes involved in eicosanoid synthesis and serve as sites for the cellular synthesis of arachidonic acid during inflammation [67]. Thus, LDs may be specific sites for transmitting intracellular inflammatory signals and also structural markers of inflammation. The previous studies suggest that LPS-induced inflammation promotes the formation of LDs in microglia, while LD-rich microglia secrete a greater amount of inflammatory mediators. However, whether LDs in glia are the hub of neuroinflammation remains to be further investigated.

ROS are potent oxidants in cells, it contributes to these LDs accumulation by triggering c-Jun-N-Terminal Kinase (JNK) and SREBP activity [68]. Mutations in several mitochondrial genes involved in Complex I proteins composition, mitochondrial fusion, and protein translation caused increased levels of intracellular ROS, which affect the homeostasis of intracellular lipid metabolism, leading to the accumulation of neutral LDs. To examine the causal relationship between oxidative stress and LDs production further, Jin and colleagues added different concentrations of hydrogen peroxide to the cell culture solution to mimic increased intracellular ROS levels. They found that LD accumulation was found in all experimental groups, but there was no significant effect on the number of LDs with different concentrations of hydrogen peroxide [63]. Increased ROS generation is one of the main characteristics of aged microglia, and LDs of microglia was significantly higher in aged than in young mice. By labeling ROS with CellROX fluorescent staining, a twofold enhancement of CellROX signal was observed in microglia with LD-high microglia compared to microglia with LD-low microglia and LDs formation inhibition significantly reduced ROS levels [69]. These results indicate that ROS may both drive and is driven by LDs accumulation in glia in a destructive cycle.

### Aging

Increased age is the largest single risk factor for the etiology of neurodegenerative diseases such as AD. Growing research shows that the accumulation of LDs in the brain may increase with age. Histological staining with BODIPY, a dye that specifically labels neutral lipids, showed that 20-month-old mice had more than four times the number of LDs within microglia in the hippocampus compared to 3-month-old mice, and the size of LDs was significantly larger [69]. In addition, an increase in LDs with age was also observed in the pia mater, cortex, and striatum of mice [70]. Similarly, within human tissues, lipid-rich microglia are more abundant in the brains of older individuals than in younger individuals [69]. Based on these findings, aging may be one of the facilitators of LD accumulation. An increase in LDs in the brain was observed by simulating the aging environment, but the underlying mechanism by which aging promotes LD accumulation is unclear, and it may be associated with age-related neuroinflammation as well as metabolism. Oil Red O-positive lipid-laden cells (LLC) were found to be widely distributed in the aging brain, Shimabukuro and colleagues detected the production of the pro-inflammatory cytokine TNF-alpha in LLC and it may participate in the age-associated neuroinflammatory processes [70]. Impaired autophagy, pro-inflammatory and senescent phenotypes are characteristic alterations of the aging brain, which could contribute to deficits in neurogenesis and synaptic plasticity. Besides that, a subtle decline in brain energy metabolism during aging [71], these pathology-related changes occurring in the aging brain may act together to cause the formation of LDs.

### Other factors

Low-density lipoprotein can cross the intima of arteries into the vessel wall, and a large amount of low-density lipoprotein cholesterol is phagocytosed by macrophages to form ‘foamy macrophages’, which were discovered to contribute to atherosclerotic lesions [72]. Upon lipid elevation, macrophages accumulate CE by binding modified lipoproteins in LDs after esterifying unesterified FC via ACAT1 [73]. The research of Lee's group proved that lipolytic products of triglyceride-rich lipoproteins (TGRL) increased the BBB transfer coefficient and induced astrocyte lipid accumulation. These results demonstrate that elevation of blood triglycerides affects the formation and accumulation of intracellular LDs either peripherally or central.

Damaged brain energy is associated with the cause and progression of neurodegenerative diseases, and nutritional deficiencies and hypoxia are common stressors in many central nervous system diseases. Exposure of astrocytes to several sources of nutritional stress, namely partial/complete nutritional deficiency, excess FFA, and L-lactate, for 24 h resulted in a significant increase in the size and/or number of LDs [74]. Consistent with the results reported by Nguyen and colleagues [57], DGAT1 and DGAT2 inhibitors reduced the accumulation of LDs in astrocytes in a nutrient-deprived state. In addition, the hypoxic environment and increased norepinephrine can also selectively promote the accumulation of LDs in astrocytes by upregulating glycogenolysis, aerobic glycolysis, and lactate production in astrocytes [75]. Defective lipolysis also contributes to intracellular lipid aggregation, for example, lack of ATGL leads to increased fat mass, and studies have demonstrated that murine ATGL-/- macrophages accumulate high amounts of TG-rich LDs [76].

## Lipid transport and transformation

### The glia-neuron coupling

The brain is a highly energy-consuming organ that efficiently utilizes a variety of energy substrates such as glucose, ketone bodies, lactate, and glutamate [77]. Lactate has been a focus of investigation as a substrate for brain energy supply. In the brain, lactate is primarily formed in astrocytes. The Astrocyte Neuron Lactate Shuttle (ANLS) Hypothesis [78] proposes that lactate is transported from glial to neurons via monocarboxylate transporters (MCTs) in Drosophila [79] and mice [80–82]. Metabolites of lactate provide the key substrate for FAs synthesis in vertebrate cells as well as in Drosophila.

During an energy crisis, FAs are stored in the LDs in the form of energy-rich TG, however, why cells store FAs under these conditions is unknown. Interestingly, there is a low content of LDs in neurons and a low capacity of neuronal mitochondria to use LDs for energy supply [83]. Contrary to neurons, astrocytes generate LDs and produce many antioxidants, and they exhibit intrinsic neuroprotective capacity by coupling metabolism with neurons [84]. To investigate whether MCTs are critical for glial LD accumulation, Liu and colleagues performed additional experiments based on the previous. By knocking down the fly homologs of MCTs or MCTs accessory protein, the researchers observed a reduction of glial LDs accumulation caused by Sicily and mitochondrial-associated regulatory factor (Marf) mutations, and the reduction of MCT levels delayed neurodegeneration [68].

ApoE is a lipid transport protein found in the peripheral and CNS, which transports and delivers cholesterol and other lipids by binding to ApoE receptors on the cell surface [85, 86]. ApoE was previously thought to be expressed mainly in astrocytes, however, a recent transcriptome analysis of human brain cells showed that ApoE transcripts are also expressed in microglia [11, 87], which links cholesterol metabolism in microglia to neurodegenerative diseases [88]. ApoE is upregulated in neurons only under oxidative stress to enhance their FA clearance, representing a possible role for apolipoproteins in regulating LD metabolism. Unlike neurons, ApoE is highly expressed in astrocytes and has three major isoforms in humans: ApoE2, ApoE3, and ApoE4. Different from the protective effect of E2 and E3, the E4 allele increases the risk of AD. Considering that astrocytes are the primary site of β-oxidation of FAs in the brain, astrocytes expressing different apolipoproteins may exhibit different capacities for FAs metabolism and LDs formation. Indeed, compared to E3 astrocytes, E4 carriers had increased expression of PLIN-2, reduced uptake and oxidation of exogenous FAs, increased oxidative oxygen consumption of endogenous FAs, and increased total volume but reduced size of LDs [89].

**Fig. 1:**
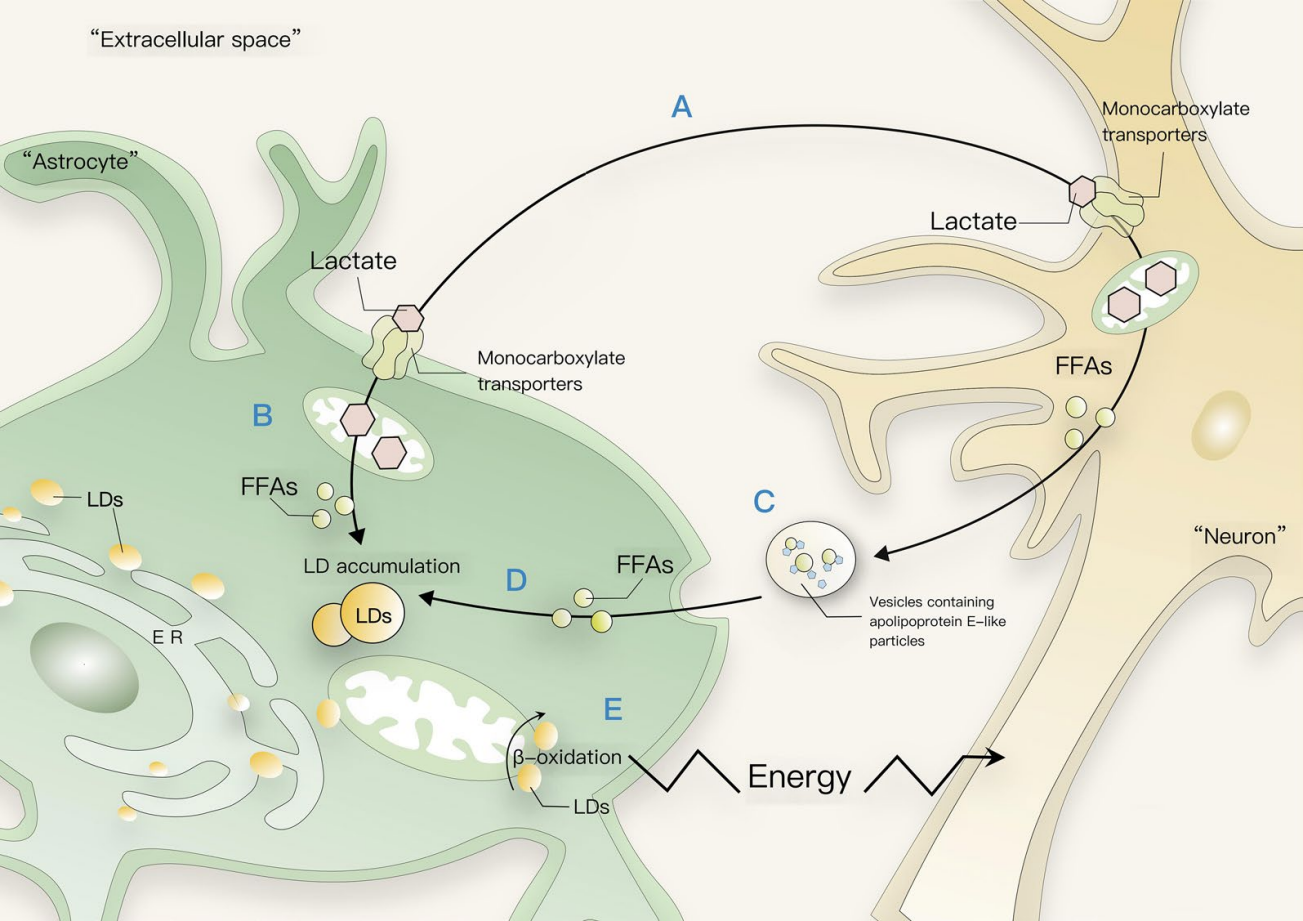
Astrocyte-neuron coupling in lipid metabolism. **A** Exogenous lactate may enter astrocytes and neurons through lactate MCTs, moreover, the reduction of MCT levels delayed neurodegeneration. **B** Glia-derived lactate is decarboxylated in neuronal mitochondria and the resulting acetyl-CoA generates FAs that are shuttled back to the glial compartment, where they accumulate in LDs. **C** Due to the low capacity of neurons to utilize LDs, it translocates excess FAs into astrocytes via vesicles containing apolipoprotein E-like particles. **D** Neuron-derived FAs are delivered to astrocyte LDs that protect astrocytes from the lipotoxicity of FAs. **E** Astrocytes can use FFAs during β-oxidation and provide the energy generated during β-oxidation to mitochondria

Magistretti’s group evokes hippocampal neurons hyperactivity with N-methyl-D-aspartate (NMDA) and observed a significant increase in FAs transfer from neurons to astrocytes by ApoE-positive lipid particles [26]. Not only are astrocytes important in the transport of FAs, but they are also the main location for β-oxidation of FAs in the brain because of their relatively high mitochondrial concentration [90, 91]. Astrocytes can both transport FAs from hyperexcitable neurons and store them in LDs, and supply the brain with energy through β-oxidation of FAs in times of starvation and stress. The RNA sequencing analysis revealed that astrocytes were found to express higher levels of oxidative stress- and lipid metabolism-related genes than neurons, and notably, genes that neutralize oxidative species superoxide radicals and are responsible for protection from free-FA toxicity were significantly upregulated in astrocytes containing LDs. Therefore, the metabolic coupling between astrocytes and neurons may be a key mechanism to alleviate neuronal excitotoxicity Fig. 1.

### LDs and mitochondrial metabolism and function in the brain

Over 95% of the brain's ATP supply is provided primarily by glucose metabolism, but the glucose reserves within the brain can meet its ATP requirements for only a few minutes, so ketone bodies and lactate are used as major alternative fuel sources when the brain is facing an energy crisis. The liver converts FAs into ketone bodies and crosses the BBB to the brain for energy supply, thus, peripheral LDs may also indirectly affect brain energy metabolism. LXRs are pivotal regulators in mammalian lipid metabolism, controlling the expression of a range of genes related to cholesterol uptake, transport, efflux, and excretion in a tissue-dependent manner. In recent studies, the nuclear receptor LXRs, which are widely distributed in the liver, is also expressed in the brain and have an important role in brain lipid homeostasis, whose absence leads to neurodegenerative pathologies [92]. These results confirm the close relationship between lipid metabolism and brain energy homeostasis. Next, we explored the potential mechanism of action between LDs and mitochondria.

Mitochondria are energy conversion centers of cells, and they are a key site for lipid oxidation and storage [93]. As the “powerhouse” of cells, mitochondria are essential for neuronal survival, alterations in mitochondrial function and metabolism promote neurodegenerative pathologies to some extent. Although neurons are primarily supplied by glucose, when glucose is limited, the brain utilizes FAs within LDs as fuel [77]. The glucose-centered view of energy metabolism in astrocytes has been challenged in recent years, fatty acid oxidation and oxidative-metabolism-related genes are up-regulated in human cortical astrocytes [94]. As a result, Eraso-Picho's group proposes that mitochondria within astrocytes may be specialized organelles.

In mammalian cells, especially in cardiac and skeletal muscle cells, LDs appears to interact closely with mitochondria. Do LDs accumulated in the brain also contact closely with mitochondria? Smolic's group labeled intracellular organelles in isolated rat brain astrocytes immunocytochemically, confirming that LDs are localized in the vicinity of mitochondria and endoplasmic reticulum, and this decrease in LDs mobility is further inhibited by metabolic stress and pressure [74]. Under starvation conditions, LDs contact mitochondria to facilitate the transport of FAs to mitochondria, thus providing fuel for oxidative phosphorylation [95]. In addition to this, mitochondria are also involved in the triacylglyceride synthesis, a process that counteracts mitochondrial fat beta-oxidation. On the other hand, Marf is required to be in contact with LDs to store cholesterol.

The contact of LDs with mitochondria is not a random event, it is regulated by several factors. MIGA2 is an outer mitochondrial membrane protein that can link mitochondria to LDs through a specific region of its C terminus [96]. Overexpression of PLIN5 was found to increase the accumulation of mitochondria with LDs, and its carboxy-terminal portion may be essential for this recruitment [97, 98], but whether specific proteins are involved in this process remains obscure. Similarly, DGAT2 can increase the contact between mitochondria and LDs [56].

Mitochondrial fusion and division, mitophagy, and transport are known as “mitochondrial dynamics”. Mitochondrial fusion and division are mediated by several related proteins, and the inactivation of fusion proteins and activation of division proteins facilitate the clearance of dysfunctional mitochondria [99]. In contrast, mitochondria anchored to LDs exhibit reduced motility and fission [97]. Lipolytic stimulation is known to promote mitochondrial-LDs contact [100], and the specific interaction between PLIN1 and MFN2(a fusion protein of the outer mitochondrial membrane) may promote mitochondria–LDs interaction by enhancing cellular responsiveness to lipolysis [101]. Consistent with this, the knockdown of MFN1 and OPA1 (a mitochondrial inner membrane fusion protein) resulted in the separation of mitochondria with LDs. Further studies revealed that the fused state of mitochondria ensures that FA is uniformly distributed throughout the mitochondria, thus achieving optimal β-oxidation within the mitochondria, while fusion-deficient mitochondria redirect FA back to LDs in response to excess non-metabolic FAs [93]. Whether the contact between LDs and mitochondria is a result of mitochondrial fusion/fission or the contact between these two affects mitochondrial dynamics, or both is still in question. The question of whether the accumulation of LDs in mitochondria alters mitochondrial dynamics and thus leads to mitochondrial dysfunction has not been studied.

Mitochondria contact LDs are termed PDM (peri-droplet mitochondria), due to the lack of a credible approach to separating PDM selectively, the role has not been well elucidated. In a recent study, Benador and colleagues effectively separate PDM from LDs in brown adipose tissue by differential centrifugation. Compared to cytoplasmic mitochondria (CM), purified PDM exhibits decreased fat oxidation capacity, low fusion-fission dynamics, and a higher ATP synthesis capacity, which may use for promoting triglyceride synthesis. The contact between mitochondria and LDs is increased in a normothermic environment, and PDM seems to be beneficial in promoting LDs storage rather than oxidation. Interestingly, the separation of PDM from LDs when thermal production or β-oxidation is activated [97].

In starvation-induced autophagy, cells recover some of their energy by degrading non-essential organelles to survive the difficulty. Autophagosomes present organelle components to lysosomes and produce FAs, which are used for the production of ATP through β-oxidation in mitochondria, however, how LDs translocate FAs to mitochondria has not been well studied. FAs produced by the degradation of membrane organelles are stored in LDs to avoid damage to cells caused by their lipotoxicity. Nguyen and his colleagues reported in their study that LDs serve as a "supply station" for mitochondrial β-oxidation on the one hand, and store FAs that are damaging to the cell membrane, on the other hand, thus providing a protective effect on mitochondria. By inhibiting DGAT1, they found that mitochondria were damaged and dysfunctional, without significant changes in ROS levels during this period, suggesting that the mitochondrial dysfunction may result from blocking the synthesis of LDs with DGAT1 inhibitors [57]. Another study indicated that mice lacking ATGL accumulate intracellular TG due to defective lipolysis, which activates the mitochondrial apoptotic pathway in macrophages, resulting in highly impaired mitochondrial function, leading to mitochondrial disruption, loss of membrane potential, reduced oxygen consumption, increased cytoplasmic Ca2 + levels and reactive oxygen species production [102]. Thus, it seems that LDs may only play a temporary role in buffering lipotoxic damage, and the mechanism of how lipid accumulation induces mitochondrial impairment or apoptosis remains to be investigated.

**Fig. 2:**
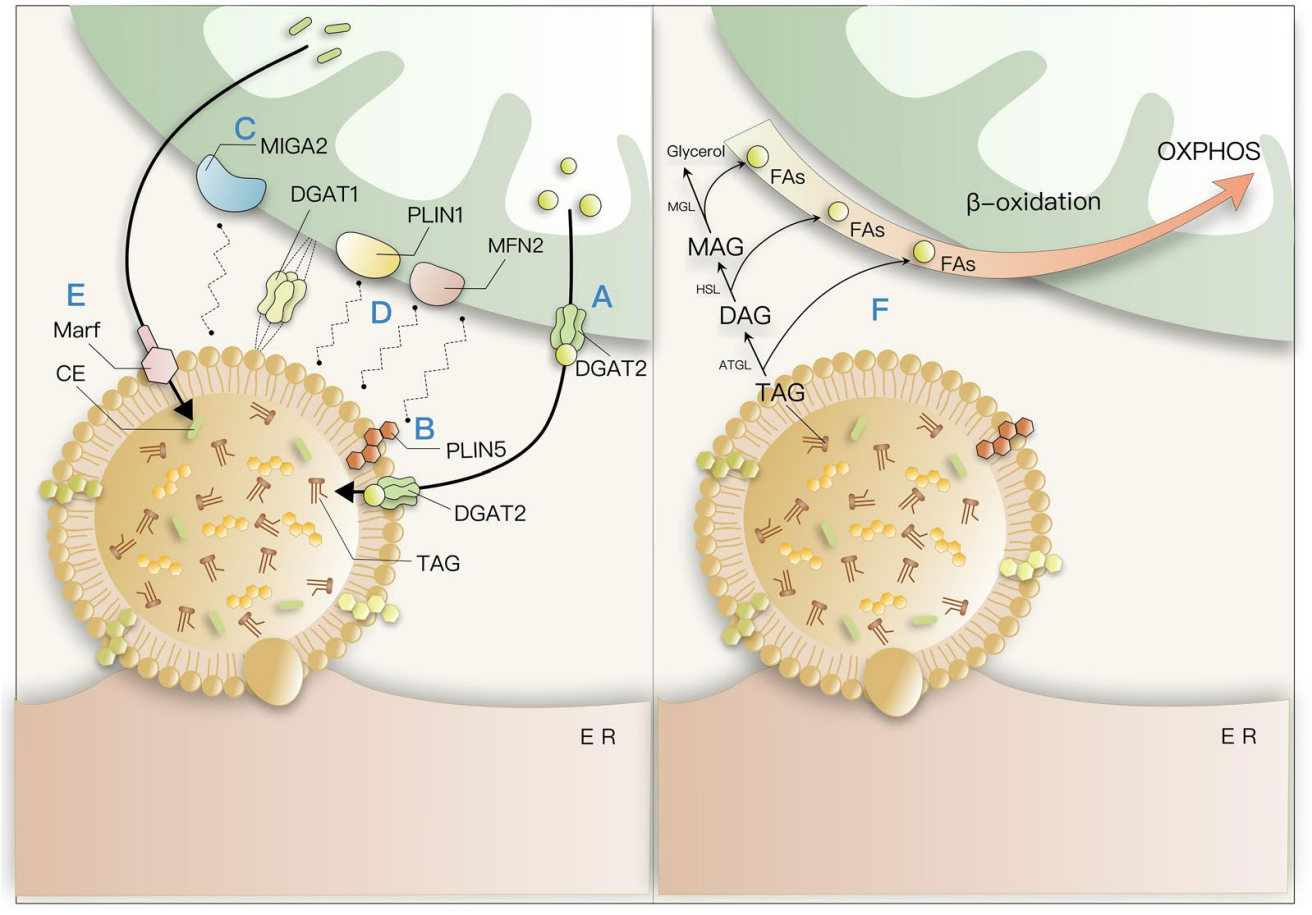
Two possible antagonistic effects of Mitochondria contact LDs. **A** DGAT2 was found to co-localize with the attachment of lipid droplet surface and mitochondria, and it may play a role in facilitating the binding of LDs to mitochondria. **B** Perilipins are LD-scaffolding proteins, PLIN-5 recruits mitochondria to the LD surface through a C-terminal region, while down-regulation of PLIN-5 expression reduces the contact of mitochondria with LDs. **C** MIGA2 is an outer mitochondrial membrane protein that can link mitochondria to LDs through a specific region of its C terminus. **D** Mitochondria anchored to LDs exhibit reduced motility and fission, the specific interaction between PLIN1 and MFN2 may promote mitochondria–LDs interaction by enhancing cellular responsiveness to lipolysis. In addition to that, the knockdown of MFN1 and OPA1 resulted in the separation of mitochondria with LDs. **E** Marf is required to be in contact with LDs to store cholesterol, neuronal cholesterol reduction induces p-Tau degradation by enhancing proteasome levels and increasing total cellular proteasome activity. **F** Underfed conditions, lipolysis is activated by LD-associated lipases, such as ATGL, HSL, and MGL, which promote the breakdown of TAG to FAs. Then, FAs are transported to mitochondria for β-oxidation

It used to be thought that oxidation and production of lipids in mitochondria will not occur simultaneously in the majority of cells, the latest research proposes that these two processes can occur simultaneously in brown adipocytes and immune cells [103]. Whether the mitochondria-LDs contact is intended to promote β-oxidation or lipid storage remains unclear. Rambold and colleagues also observed amplification of LDs around mitochondria, but they focused more on the effect of LDs' contact with mitochondria on β-oxidation. Since the knockout of Mfn1 and Opa1 resulted in impaired fatty acid oxidation, they proposed that facilitating the transport of FAs to mitochondria for β-oxidation may be one of the functions of PDM [93]. This view seems to be corroborated by the Marschallinger group's experiments that genes associated with the ‘fatty acid β-oxidation’ pathway are significantly upregulated in LD-rich microglia Fig. 2.

## Lipid accumulation in some common neurodegenerative diseases

### AD

Alzheimer's disease is the most common neurodegenerative disease worldwide, hyperphosphorylated tau and Aβ plaque are thought to be the two major pathological features of AD [104]. The presence of adipose inclusions in microglia was noted by Alois Alzheimer in 1907 when he first described the case of AD and documented it in his paper [105]. Most of the research on AD has been centered on Tau and A amyloid plaques, and the role of LD accumulation in the lesioned brain has only come to the forefront in recent years. CE is the storage product of excess cholesterol and can be integrated into the LD core during normal LDs biogenesis. The brain produces its cholesterol, current research suggests that excess brain cholesterol regulates Tau and Aβ deposition independently, and neuronal cholesterol reduction induces pTau degradation by enhancing proteasome levels and increasing total cellular proteasome activity [106]. Intracellular cholesterol accumulation disrupts autophagic flux and leads to reduced Aβ clearance from defective mitochondria [107]. Notably, many publications suggest that Aβ oligomers promote increased neuronal cytoplasmic Ca2 + concentrations [108, 109], and the calcium (Ca2 +) hypothesis of AD states that mishandling of calcium signals in neurons is a key event triggering synaptic dysfunction and neurodegeneration. SP has been shown to contribute to the pathological process of AD, and ceramide promotes the production and accumulation of Aβ by stabilizing beta-site amyloid precursor protein-cleaving enzyme 1 (BACE-1) [110]. Interestingly, the accumulation of Aβ stimulates sphingolipid hydrolysis [111], thereby increasing ceramide levels in AD. In addition, ceramide-rich exosomes are involved in plaque formation, and in vitro experiments show that astrocyte-derived exosomes accelerate Aβ 42 accumulation and prevent its glial clearance [112].

Numerous studies have shown that the ApoE4 allele is a strong genetic factor in AD and regulates the pathological progression of the disease through various pathways such as energy metabolism, lipid transport, and synaptic plasticity [113]. ApoE4 significantly enhances tau-mediated neurodegeneration, whereas ApoE4 deletion is protective [114]. ApoE4 exhibits lower autophagic flux compared to ApoE3, the apoE4-Aβ complex is less stable than ApoE3-Aβ, and ApoE4 has a lower facilitation effect on Aβ clearance than apoE3 [115, 116], which may be one of the factors that promote the progression of AD. Similarly, in peripheral diseases such as atherosclerosis, the ineffective transport of lipids by ApoE4 may contribute to lipid accumulation and increased lipid peroxidation [117, 118].

### PD

PD is characterized by the progressive loss or degeneration of the dopaminergic (DA) neurons in the substantia nigra and the accumulation of a-synuclein in DA neurons. Lewy bodies are the intracerebral hallmark of PD patients, the misfolded a-synuclein enters it and aggregates abnormally [119]. Lipids have been found in synuclein-containing Lewy bodies purified from human PD brains [120], Cole suggests that lipolysis of LDs may contribute to PD, and the wild type of α-synuclein protein binds to LDs phospholipid surface for slowing lipolysis of LDs [121]. The PD mutant synucleins A30P showed no LD binding, and the A53T mutant, although it does not prevent LD binding, Cole and colleagues propose that it no longer slows down triglyceride hydrolysis as effectively as wild type synuclein as the conformation of the protein has altered [121]. Fanning found that LDs are protective against a-synuclein toxicity in neurons. They also found that α-synuclein excess alters neutral lipid homeostasis in human neurons, these lipid alterations at least caused toxicity partially. Fanning and colleagues suppress the oleic acid generation to reduce a-synuclein toxicity by partially inhibiting SCD, providing a rational therapeutic approach to PD.

High levels of cholesterol and its oxidized cholesterol products (oxysterols) trigger several pathological pathways such as oxidation, inflammation, and cell death, promoting the accumulation of α-synuclein and contributing to the pathophysiology of PD [122]. Alterations in ceramide metabolism also occur in Parkinson's disease. Mutations in CerS1, a major neuron-specific neuron that synthesizes 18-carbon fatty acyl (C18) ceramides, lead to elevated long-chain base (LCB) substrates and decreased C18 ceramides and their derivatives in the brain, resulting in neurodegeneration in mice and myoclonic epilepsy in human dementia [123]. A lipidomics study reported that analysis of PD brain samples detected elevated levels of several ceramide species, including monohexose ceramide, lactose ceramide, and sphingomyelin [124]. The glucocerebrosidase-1 (GBA1) gene encodes glucocerebrosidase (GCase), which degrades the glycolipid glucose ceramide (GlcCer) to glucose and ceramide, and loss of GCase activity due to GBA mutations may be one of the major genetic contributors to the development of PD [125]. In addition, insufficient GCase levels and reduced activity can also increase α-synuclein accumulation by affecting protein phosphate 2A (PP2A) activity [126].

### ALS

ALS is the most common motor neuron disease, in addition to the imbalance of energy metabolism and cell stress, clearance of misfolded proteins plays an important role in ALS. When misfolded proteins accumulate and cause endoplasmic reticulum stress, a specific mechanism is required to transport them to the ubiquitin–proteasome system for degradation, and this proteasome-mediated degradation is dependent on LDs. Mutations in the human VAMP-associated protein B (hVAPB)-induced neurotoxicity pathway regulators were mainly enriched for proteins related to LD dynamics [127] and controlled the degradation of misfolded proteins via binding TTC39B [128]. Compared to the wild-type of hVAPB, the ALS8 protein hVAPB bind TTC39B more weakly. Thus, LD biogenesis or deficiency in its clearance of misfolded protein function plays an important role in hVAPB-mediated ALS.

## Conclusion and outlook

The impact of impaired lipid metabolism or pathological factors in the brain resulting in lipid accumulation and storage on neurodegenerative pathologies has received growing attention. Despite a growing number of studies describing the potential link between glial cell lipid metabolism and storage on brain development and neurodegeneration, many fundamental questions remain unresolved. What are the critical values for lipid accumulation and storage in different brain cell types? The formation of LDs in neuroglia is an active or passive process? What are the initiating factors for lipid metabolism by glial coupled with neurons? Is the release of inflammatory factors an upstream mechanism or a downstream effect of lipid-rich microglia? In fact, we are only beginning to dissect the role of neuroglial lipid metabolism in the control of brain physiology and pathology.

The accumulation of lipids in cells is often associated with energy metabolism, and other functions have been much less studied compared to the energy storage role of LDs. Although Alzheimer first documented the existence of LDs in the brain in his paper, researchers over the next several decades seem to have viewed them only as a lipid storage organelle. LD research is booming, in the periphery, LDs have been shown to play diverse and important roles in lipid metabolism and protein handling in addition to energy storage function. Lipid accumulation in glial cells is now recognized in neurodegenerative diseases, which has been verified and proven to be Pathologically important. In the CNS, LDs are predominantly distributed within glial cells, and certain LD-containing glial cells appear to exhibit distinctive phenotypes associated with neurodegeneration such as reduced phagocytosis, elevated ROS levels, and enhanced inflammatory factor secretion [69].

Lipid metabolism affects brain energy metabolism in direct and indirect ways. Impaired brain energy metabolism drives neurodegeneration, and when glucose metabolism is restricted, ketone bodies produced by the liver and lactic acid produced by muscles become important replacement fuels for the brain [129]. Due to the presence of the BBB, LDs are unable to enter the brain directly, nevertheless, ketone bodies and lactate metabolism can act as products of LDs and substrate providers for FAs production in the brain respectively, which corroborates that peripheral LDs also have an indirect effect on brain energy metabolism. LDs within astrocytes exhibit poor mobility around mitochondria, and previous studies have demonstrated that PDM may have two opposing roles in lipid metabolism: promoting β-oxidation and facilitating lipid synthesis. Despite the opposing views presented by researchers on this topic, there is perhaps no conflict between these two roles between mitochondria and LDs. In a state of nutrient deprivation, LDs in astrocytes act as energy storage organelles supplying FAs to neurons to tide them over, and similarly, β-oxidation related genes are upregulated in the Grn-dementia mouse model. However, the study of Marschallinger 's group focused on the unique phenotype of lipid-rich microglia, whether imbalances in energy metabolism occur in models of dementia has not been explored. On the other hand, neurons store excess FA in LDs, which may be a potential energy-protective mechanism besides avoiding lipotoxicity. The synthesis of lipids in mitochondria seems to be more of a protective mechanism. DGAT1-dependent LDs biogenesis shields mitochondria from FAs produced by autophagy [57]. Impaired mitochondrial fatty acid synthesis function also leads to abnormal mitochondrial morphology and decreased respiratory chain enzyme activity, ultimately leading to mitochondrial enoyl reductase protein-associated neurodegeneration [130]. Exploring the role of mitochondria and LDs in different brain cells helps to better understand the role of LDs in cells. Meanwhile, it is essential to understand the modulators of the interaction between mitochondria and LDs for the therapy of neurodegenerative diseases.

Astrocytes are closely related to lipid metabolism, they provide a lipid buffering system to mitigate lipotoxic neuronal damage and exhibit intrinsic energy supply and neuroprotective capacity by coupling metabolism with neurons, while microglia sense saturated FAs and orchestrate inflammation and neuronal stress in the mediobasal hypothalamus [131]. In the above article, it was mentioned that LD-rich microglia have increased secretion of pro-inflammatory factors and upregulated ROS levels, while another study showed that LDs within glial cells act as antioxidant organelles to protect neural stem cells [132]. The role of LDs seems to depend greatly on the type of cell and the composition of the LD, docosahexaenoic acid (DHA), an omega-3 polyunsaturated fatty acid attenuates microglial cell inflammatory response by remodeling LDs and altering their functional interplay with mitochondria and other associated organelles [133, 134], which may provide a potential therapeutic target for neurodegenerative diseases. The composition and localization of LDs vary in different types of neurodegenerative diseases and thus may have a protective or damaging effect on the brain. How to maximize the protective effect of LDs while minimizing their damaging effect is the key to mitigating the disease process and even curing it.

In conclusion, the extent to which lipid metabolism is altered and cells generate LDs to control many key brain functions is remarkable. The balance of lipid metabolism in the brain, including cholesterol and SP, is inextricably linked to neuroglia. The LDs in neuroglia is not merely a simple lipid storage cell in the diseased brain, it plays an important role in the onset and development of neurodegenerative diseases in terms of oxidative stress, neuroinflammation, and energy metabolism. Further understanding of how lipid metabolic pathways are integrated into glial cells and the brain may provide new insights into the pathogenesis of some neurodegenerative diseases. Equally important, exploring the functional phenotype of different fine different LD-containing neuroglia should be a cornerstone of trials attempting to delay the onset and progression of neurodegenerative diseases.

## Data Availability

Not applicable.

## Abbreviations

LDs: Lipid droplets
CE: Cholesteryl esters
BBB: The blood–brain barrier
SREBP: Sterol regulatory element-binding protein
EAE: Encephalomyelitis
TREM2: Triggering receptor expressed on myeloid cell 2
ACAT: Acyltransferase
FAs: Fatty acids
FFAs: Free fatty acids
SP: Sphingolipids
S1P: Sph-1-phosphate
nSMase: Neutral Sphingomyelinase-2
CerS: Ceramide synthase
MS: Multiple sclerosis
TG: Triacylglycerols
SEs: Sterol esters
DGAT: Diacylglycerol acyltransferase
PLINs: Perilipins
ATGL: Adipose triglyceride lipase
HSL: Hormone-sensitive lipase
MGL: Monoglyceride lipase
DAG: Diacylglycerol
MAG: Monoacylglycerol
ATP: Adenosine triphosphate
LDAM: LD accumulating microglia
LPS: Lipopolysaccharide
JNK: Jun-N-Terminal Kinase
LLC: Lipid-laden cells
FC: Free cholester
TGRL: Triglyceride-rich lipoproteins
ANLS: Astrocyte Neuron Lactate Shuttle
MCTs: Monocarboxylate transporters
AD: Alzheimer's disease
PD: Parkinson's disease
ALS: Amyotrophic lateral sclerosis
HD: Huntington’s disease
NMDA: N-methyl-D-aspartate
ApoE: Apolipoprotein E
CNS: Central nervous system
Aβ: β-Amyloid
LXRs: The liver X receptors
Marf: Mitochondrial-associated regulatory factor
PDM: Peri-droplet mitochondria
CM: Cytoplasmic mitochondria
DA: Dopaminergic
SCD: Stearoyl-CoA-desaturase
C18: 18-Carbon fatty acyl
hVAPB: Human VAMP-associated protein B
DHA: Docosahexaenoic acid

## References

[1] Cermenati G (2015). Lipids in the nervous system: from biochemistry and molecular biology to patho-physiology. Biochim Biophys Acta.

[2] Korade Z, Kenworthy AK (2008). Lipid rafts, cholesterol, and the brain. Neuropharmacology.

[3] Genaro-Mattos TC (2019). Cholesterol biosynthesis and uptake in developing neurons. ACS Chem Neurosci.

[4] Linetti A (2010). Cholesterol reduction impairs exocytosis of synaptic vesicles. J Cell Sci.

[5] Boyles JK (1989). A role for apolipoprotein E, apolipoprotein A-I, and low density lipoprotein receptors in cholesterol transport during regeneration and remyelination of the rat sciatic nerve. J Clin Invest.

[6] Nieweg K (2009). Marked differences in cholesterol synthesis between neurons and glial cells from postnatal rats产后大鼠神经元和神经胶质细胞之间胆固醇合成的显着差异. J Neurochem.

[7] Koudinov AR, Koudinova NV (2002). Cholesterol's role in synapse formation. Science.

[8] Morell P, Jurevics H (1996). Origin of cholesterol in myelin. Neurochem Res.

[9] Mauch DH (2001). CNS synaptogenesis promoted by glia-derived cholesterol. Science.

[10] Chang TY (2006). Cholesterol sensing, trafficking, and esterification. Annu Rev Cell Dev Biol.

[11] Puglielli L (2001). Acyl-coenzyme a: cholesterol acyltransferase modulates the generation of the amyloid beta-peptide. Nat Cell Biol.

[12] Bryleva EY (2010). ACAT1 gene ablation increases 24(S)-hydroxycholesterol content in the brain and ameliorates amyloid pathology in mice with AD. Proc Natl Acad Sci USA.

[13] Astudillo L (2015). Human genetic disorders of sphingolipid biosynthesis. J Inherit Metab Dis.

[14] Bartke N, Hannun YA (2009). Bioactive sphingolipids: metabolism and function. J Lipid Res.

[15] Olsen ASB, Faergeman NJ (2017). Sphingolipids: membrane microdomains in brain development, function and neurological diseases. Open Biol.

[16] Mullen TD (2012). Ceramide synthases at the centre of sphingolipid metabolism and biology. Biochem J.

[17] Satoi H (2005). Astroglial expression of ceramide in Alzheimer's disease brains: a role during neuronal apoptosis. Neuroscience.

[18] Filippov V (2012). Increased ceramide in brains with Alzheimer's and other neurodegenerative diseases. J Alzheimers Dis.

[19] Senkal CE (2017). Ceramide is metabolized to acylceramide and stored in lipid droplets. Cell Metab.

[20] Yuyama K (2012). Sphingolipid-modulated exosome secretion promotes clearance of amyloid-β by microglia. J Biol Chem.

[21] Hait NC (2009). Regulation of histone acetylation in the nucleus by sphingosine-1-phosphate. Science.

[22] Spiegel S, Milstien S (2011). The outs and the ins of sphingosine-1-phosphate in immunity. Nat Rev Immunol.

[23] Karunakaran I (2019). Neural sphingosine 1-phosphate accumulation activates microglia and links impaired autophagy and inflammation. Glia.

[24] Dhopeshwarkar GA, Mead JF (1970). Fatty acid uptake by the brain. 3. Incorporation of (1–14C)oleic acid into the adult rat brain. Biochim Biophys Acta.

[25] Ebert D (2003). Energy contribution of octanoate to intact rat brain metabolism measured by 13C nuclear magnetic resonance spectroscopy. J Neurosci.

[26] Ioannou MS (2019). Neuron-astrocyte metabolic coupling protects against activity-induced fatty acid toxicity. Cell.

[27] Butterfield DA (1801). (2010) Involvements of the lipid peroxidation product, HNE, in the pathogenesis and progression of Alzheimer's disease. Biochim Biophys Acta.

[28] Lee J (2011). Modulation of lipid peroxidation and mitochondrial function improves neuropathology in Huntington's disease mice. Acta Neuropathol.

[29] Ruiperez V (2010). Alpha-synuclein, lipids and Parkinson's disease. Prog Lipid Res.

[30] Ferrante RJ (1997). Evidence of increased oxidative damage in both sporadic and familial amyotrophic lateral sclerosis. J Neurochem.

[31] Sultana R (2013). Lipid peroxidation triggers neurodegeneration: a redox proteomics view into the Alzheimer disease brain. Free Radic Biol Med.

[32] Pfrieger FW (2003). Outsourcing in the brain: do neurons depend on cholesterol delivery by astrocytes?. BioEssays.

[33] van Deijk AF (2017). Astrocyte lipid metabolism is critical for synapse development and function in vivo. Glia.

[34] Ferris HA (2017). Loss of astrocyte cholesterol synthesis disrupts neuronal function and alters whole-body metabolism. Proc Natl Acad Sci U S A.

[35] Karten B (2006). Expression of ABCG1, but not ABCA1, correlates with cholesterol release by cerebellar astroglia. J Biol Chem.

[36] de Wit NM (2019). Astrocytic ceramide as possible indicator of neuroinflammation. J Neuroinflammation.

[37] Chao CC (2019). Metabolic control of astrocyte pathogenic activity via cPLA2-MAVS. Cell.

[38] Choi JW (2011). FTY720 (fingolimod) efficacy in an animal model of multiple sclerosis requires astrocyte sphingosine 1-phosphate receptor 1 (S1P1) modulation. Proc Natl Acad Sci USA.

[39] Hickman SE (2013). The microglial sensome revealed by direct RNA sequencing. Nat Neurosci.

[40] Grajchen E (2020). CD36-mediated uptake of myelin debris by macrophages and microglia reduces neuroinflammation. J Neuroinflammation.

[41] Nugent AA (2020). TREM2 regulates microglial cholesterol metabolism upon chronic phagocytic challenge. Neuron.

[42] Yeh FL (2016). TREM2 binds to apolipoproteins, including APOE and CLU/APOJ, and thereby facilitates uptake of amyloid-beta by microglia. Neuron.

[43] Gaultier A (2009). Low-density lipoprotein receptor-related protein 1 is an essential receptor for myelin phagocytosis. J Cell Sci.

[44] Bogie JF (2012). Myelin-derived lipids modulate macrophage activity by liver X receptor activation. PLoS ONE.

[45] Liang Y (2004). A liver X receptor and retinoid X receptor heterodimer mediates apolipoprotein E expression, secretion and cholesterol homeostasis in astrocytes. J Neurochem.

[46] Jain A, Holthuis JCM (1864). (2017) Membrane contact sites, ancient and central hubs of cellular lipid logistics. Biochim Biophys Acta Mol Cell Res.

[47] Cantuti-Castelvetri L (2018). Defective cholesterol clearance limits remyelination in the aged central nervous system. Science.

[48] Bogie JFJ (2020). Stearoyl-CoA desaturase-1 impairs the reparative properties of macrophages and microglia in the brain. J Exp Med.

[49] Hendrickx DA (2014). Enhanced uptake of multiple sclerosis-derived myelin by THP-1 macrophages and primary human microglia. J Neuroinflammation.

[50] Lynch JR (2003). APOE genotype and an ApoE-mimetic peptide modify the systemic and central nervous system inflammatory response. J Biol Chem.

[51] Bergner CG (2019). Microglia damage precedes major myelin breakdown in X-linked adrenoleukodystrophy and metachromatic leukodystrophy. Glia.

[52] Valachovic M (2016). Squalene is lipotoxic to yeast cells defective in lipid droplet biogenesis. Biochem Biophys Res Commun.

[53] Schmidt C (2013). Analysis of yeast lipid droplet proteome and lipidome. Methods Cell Biol.

[54] Weiss SB (1960). The enzymatic synthesis of triglycerides. J Biol Chem.

[55] Nguyen TB, Olzmann JA (2017). Lipid droplets and lipotoxicity during autophagy. Autophagy.

[56] Stone SJ (2009). The endoplasmic reticulum enzyme DGAT2 is found in mitochondria-associated membranes and has a mitochondrial targeting signal that promotes its association with mitochondria. J Biol Chem.

[57] Nguyen TB (2017). DGAT1-dependent lipid droplet biogenesis protects mitochondrial function during starvation-induced autophagy. Dev Cell.

[58] Olzmann JA, Carvalho P (2019). Dynamics and functions of lipid droplets. Nat Rev Mol Cell Biol.

[59] Gross DA (2011). Direct binding of triglyceride to fat storage-inducing transmembrane proteins 1 and 2 is important for lipid droplet formation. Proc Natl Acad Sci USA.

[60] Murphy DJ (2012). The dynamic roles of intracellular lipid droplets: from archaea to mammals. Protoplasma.

[61] Walther TC, Farese RV (2012). Lipid droplets and cellular lipid metabolism. Annu Rev Biochem.

[62] Sztalryd C, Brasaemle DL (1862). (2017) The perilipin family of lipid droplet proteins: Gatekeepers of intracellular lipolysis. Biochim Biophys Acta Mol Cell Biol Lipids.

[63] Jin Y (2018). Reactive oxygen species induces lipid droplet accumulation in HepG2 cells by increasing perilipin 2 expression. Int J Mol Sci.

[64] Kounakis K (2019). Emerging roles of lipophagy in health and disease. Front Cell Dev Biol.

[65] Ritzel RM (2015). Age- and location-related changes in microglial function. Neurobiol Aging.

[66] Yu W (2000). Phosphatidylinositide 3-kinase localizes to cytoplasmic lipid bodies in human polymorphonuclear leukocytes and other myeloid-derived cells. Blood.

[67] Bozza PT, Viola JP (2010). Lipid droplets in inflammation and cancer. Prostaglandins Leukot Essent Fatty Acids.

[68] Liu L (2015). Glial lipid droplets and ROS induced by mitochondrial defects promote neurodegeneration. Cell.

[69] Marschallinger J (2020). Lipid-droplet-accumulating microglia represent a dysfunctional and proinflammatory state in the aging brain. Nat Neurosci.

[70] Shimabukuro MK (2016). Lipid-laden cells differentially distributed in the aging brain are functionally active and correspond to distinct phenotypes. Sci Rep.

[71] Yin F (2016). Energy metabolism and inflammation in brain aging and Alzheimer's disease. Free Radical Biol Med.

[72] Fowler SD (1985). Foam cells and atherogenesis. Ann NY Acad Sci.

[73] Brown MS (1980). The cholesteryl ester cycle in macrophage foam cells. Continual hydrolysis and re-esterification of cytoplasmic cholesteryl esters. J Biol Chem.

[74] Smolic T (2021). Astrocytes in stress accumulate lipid droplets. Glia.

[75] Dienel GA, Cruz NF (2016). Aerobic glycolysis during brain activation: adrenergic regulation and influence of norepinephrine on astrocytic metabolism. J Neurochem.

[76] Chandak PG (2010). Efficient phagocytosis requires triacylglycerol hydrolysis by adipose triglyceride lipase. J Biol Chem.

[77] Zielke HR (2009). Direct measurement of oxidative metabolism in the living brain by microdialysis: a review. J Neurochem.

[78] Pellerin L, Magistretti PJ (1994). Glutamate uptake into astrocytes stimulates aerobic glycolysis: a mechanism coupling neuronal activity to glucose utilization. Proc Natl Acad Sci USA.

[79] Volkenhoff A (2015). Glial glycolysis is essential for neuronal survival in drosophila. Cell Metab.

[80] Funfschilling U (2012). Glycolytic oligodendrocytes maintain myelin and long-term axonal integrity. Nature.

[81] Lee Y (2012). Oligodendroglia metabolically support axons and contribute to neurodegeneration. Nature.

[82] Machler P (2016). In vivo evidence for a lactate gradient from astrocytes to neurons. Cell Metab.

[83] Schonfeld P, Reiser G (2017). Brain energy metabolism spurns fatty acids as fuel due to their inherent mitotoxicity and potential capacity to unleash neurodegeneration. Neurochem Int.

[84] Belanger M, Magistretti PJ (2009). The role of astroglia in neuroprotection. Dialogues Clin Neurosci.

[85] Mahley RW, Rall SC (2000). Apolipoprotein E: far more than a lipid transport protein. Annu Rev Genomics Hum Genet.

[86] Mahley RW (1988). Apolipoprotein E: cholesterol transport protein with expanding role in cell biology. Science.

[87] Xu Q (2006). Profile and regulation of apolipoprotein E (ApoE) expression in the CNS in mice with targeting of green fluorescent protein gene to the ApoE locus. J Neurosci.

[88] Keren-Shaul H (2017). A unique microglia type associated with restricting development of Alzheimer's disease. Cell.

[89] Farmer BC (2019). Apolipoprotein E4 alters astrocyte fatty acid metabolism and lipid droplet formation. Cells.

[90] Edmond J (1987). Capacity for substrate utilization in oxidative metabolism by neurons, astrocytes, and oligodendrocytes from developing brain in primary culture. J Neurosci Res.

[91] Lovatt D (2007). The transcriptome and metabolic gene signature of protoplasmic astrocytes in the adult murine cortex. J Neurosci.

[92] Hong C, Tontonoz P (2014). Liver X receptors in lipid metabolism: opportunities for drug discovery. Nat Rev Drug Discovery.

[93] Rambold AS (2015). Fatty acid trafficking in starved cells: regulation by lipid droplet lipolysis, autophagy, and mitochondrial fusion dynamics. Dev Cell.

[94] Eraso-Pichot A (2018). GSEA of mouse and human mitochondriomes reveals fatty acid oxidation in astrocytes. Glia.

[95] Bosch M (2020). Lipid droplets, bioenergetic fluxes, and metabolic flexibility. Semin Cell Dev Biol.

[96] Freyre CAC (2019). MIGA2 links mitochondria, the ER, and lipid droplets and promotes de novo lipogenesis in adipocytes. Mol Cell.

[97] Benador IY (2018). Mitochondria bound to lipid droplets have unique bioenergetics, composition, and dynamics that support lipid droplet expansion. Cell Metab.

[98] Wang H (2011). Perilipin 5, a lipid droplet-associated protein, provides physical and metabolic linkage to mitochondria. J Lipid Res.

[99] Twig G (2008). Fission and selective fusion govern mitochondrial segregation and elimination by autophagy. EMBO J.

[100] Yu J (1853). (2015) Lipid droplet remodeling and interaction with mitochondria in mouse brown adipose tissue during cold treatment. Biochim Biophys Acta.

[101] Boutant M (2017). Mfn2 is critical for brown adipose tissue thermogenic function. EMBO J.

[102] Aflaki E (2011). Triacylglycerol accumulation activates the mitochondrial apoptosis pathway in macrophages. J Biol Chem.

[103] Mottillo EP (2014). Coupling of lipolysis and de novo lipogenesis in brown, beige, and white adipose tissues during chronic beta3-adrenergic receptor activation. J Lipid Res.

[104] Bird TD. Alzheimer Disease Overview. In GeneReviews((R)) (Adam, M.P. et al. eds). 1993

[105] Alzheimer A (1995). An English translation of Alzheimer's 1907 paper, "Uber eine eigenartige Erkankung der Hirnrinde". Clin Anat.

[106] van der Kant R (2019). Cholesterol metabolism is a druggable axis that independently regulates Tau and Amyloid-β in iPSC-derived Alzheimer's Disease neurons. Cell Stem Cell.

[107] Roca-Agujetas V (2021). Cholesterol alters mitophagy by impairing optineurin recruitment and lysosomal clearance in Alzheimer's disease. Mol Neurodegener.

[108] Demuro A (2005). Calcium dysregulation and membrane disruption as a ubiquitous neurotoxic mechanism of soluble amyloid oligomers. J Biol Chem.

[109] Demuro A (2010). Calcium signaling and amyloid toxicity in Alzheimer disease. J Biol Chem.

[110] Puglielli L (2003). Ceramide stabilizes beta-site amyloid precursor protein-cleaving enzyme 1 and promotes amyloid beta-peptide biogenesis. J Biol Chem.

[111] Haughey NJ (1801). (2010) Roles for dysfunctional sphingolipid metabolism in Alzheimer's disease neuropathogenesis. Biochim Biophys Acta.

[112] Dinkins MB (2016). Neutral sphingomyelinase-2 deficiency ameliorates Alzheimer's disease pathology and improves cognition in the 5XFAD mouse. J Neurosci.

[113] Zhao N (2018). Apolipoprotein E, receptors, and modulation of Alzheimer's disease. Biol Psychiatry.

[114] Shi Y (2017). ApoE4 markedly exacerbates tau-mediated neurodegeneration in a mouse model of tauopathy. Nature.

[115] Reger MA (2008). Intranasal insulin administration dose-dependently modulates verbal memory and plasma amyloid-beta in memory-impaired older adults. J Alzheimers Dis.

[116] Claxton A (2015). Long-acting intranasal insulin detemir improves cognition for adults with mild cognitive impairment or early-stage Alzheimer's disease dementia. J Alzheimers Dis.

[117] Jofre-Monseny L (2008). Impact of apoE genotype on oxidative stress, inflammation and disease risk. Mol Nutr Food Res.

[118] Simonovitch S (2016). Impaired Autophagy in APOE4 Astrocytes. J Alzheimer's Dis.

[119] Dickson DW (2008). Evidence that incidental Lewy body disease is pre-symptomatic Parkinson's disease. Acta Neuropathol.

[120] Gai WP (2000). In situ and in vitro study of colocalization and segregation of alpha-synuclein, ubiquitin, and lipids in Lewy bodies. Exp Neurol.

[121] Cole NB (2002). Lipid droplet binding and oligomerization properties of the Parkinson's disease protein alpha-synuclein. J Biol Chem.

[122] Doria M (2016). Contribution of cholesterol and oxysterols to the pathophysiology of Parkinson's disease. Free Radic Biol Med.

[123] Spassieva SD (2016). Ectopic expression of ceramide synthase 2 in neurons suppresses neurodegeneration induced by ceramide synthase 1 deficiency. Proc Natl Acad Sci USA.

[124] Abbott SK (2014). Altered ceramide acyl chain length and ceramide synthase gene expression in Parkinson’s disease. Mov Disord.

[125] Pchelina S (2017). Oligomeric α-synuclein and glucocerebrosidase activity levels in GBA-associated Parkinson's disease. Neurosci Lett.

[126] Rocha EM (2018). Alpha-synuclein: Pathology, mitochondrial dysfunction and neuroinflammation in Parkinson's disease. Neurobiol Dis.

[127] Sanhueza M (2015). Network analyses reveal novel aspects of ALS pathogenesis. PLoS Genet.

[128] Huttlin EL (2015). The BioPlex network: a systematic exploration of the human interactome. Cell.

[129] Cunnane SC (2016). Can ketones help rescue brain fuel supply in later life? Implications for cognitive health during aging and the treatment of Alzheimer's disease. Front Mol Neurosci.

[130] Nair RR (2018). Impaired mitochondrial fatty acid synthesis leads to neurodegeneration in mice. J Neurosci.

[131] Valdearcos M (2014). Microglia dictate the impact of saturated fat consumption on hypothalamic inflammation and neuronal function. Cell Rep.

[132] Bailey AP (2015). Antioxidant role for lipid droplets in a stem cell niche of drosophila. Cell.

[133] Tremblay ME (2016). Remodeling of lipid bodies by docosahexaenoic acid in activated microglial cells. J Neuroinflammation.

[134] Bazan NG (2018). Docosanoids and elovanoids from omega-3 fatty acids are pro-homeostatic modulators of inflammatory responses, cell damage and neuroprotection. Mol Aspects Med.

